# Development of a Community-Based Network to Promote Anti-Drug Messaging and Identify Hidden Drug Abusers in Hong Kong

**DOI:** 10.3390/ijerph191811544

**Published:** 2022-09-14

**Authors:** Ka Yan Ho, Ka Wai Katherine Lam, Edmond Tak Fai Tong, Shara Ho, Cynthia Sau Ting Wu, Man Nok Tong, Lai Ngo Tang, Yim Wah Mak

**Affiliations:** 1 School of Nursing, Hong Kong Polytechnic University, Hong Kong SAR, China; 2 Community Health Organisation for Intervention, Care and Empowerment, Hong Kong SAR, China

**Keywords:** hidden drug abuse, community-based network, anti-drug ambassadors, referral, health promotion

## Abstract

Developing a community-based network by training peers as anti-drug ambassadors (ADAs) is a feasible strategy to identify hidden drug abusers. The Ask, Warn, Advise, Refer and Do-it-again (AWARD) model of smoking cessation is useful for enhancing people’s confidence in making referrals to anti-drug services. This study evaluated the effectiveness of such a network by examining the change in knowledge, attitudes and practices (KAP) of 198 ADAs aged 13–18 before and after six months of our training. A one-group pre-test and repeated post-test design was used. One-way repeated-measures analysis of variance was applied to assess the changes in KAP, with *p*-values adjusted by Bonferroni correction. The results showed that the ADAs statistically significantly improved their KAP regarding drug abuse at the six-month follow-up compared to baseline. All ADAs who knew drug abusers (*n* = 3) had referred them to services based on the AWARD model. A total of 154 anti-drug abuse activities were conducted, reaching 4561 people. Based on the results, we concluded that the community-based network was effective in improving the KAP of ADAs regarding drug abuse, as well as referring hidden drug abusers. Future studies should consider implementing the network on a larger scale, thus maximizing its anti-drug capacity.

## 1. Introduction

Drug abuse refers to the hazardous use of psychoactive substances [1]. The World Health Organization has estimated that approximately 275 million people used an illicit drug at least once in 2016, with an annual prevalence of 5.6% [2]. Drug abuse leads to both short-term and long-term health consequences, depending on the drug type [3]. In the short term, it leads to dramatic changes in appetite, wakefulness, heart rate and blood pressure [3], while in the long run, drug abuse causes irreversible damage to most organs, resulting in various chronic health problems such as emphysema, heart attacks, cancer, hepatitis, infertility, psychosis and depression [3]. The health consequences of drug abuse are cumulative and result in a significant cost to the healthcare system [4].

Drug abuse is a serious problem among young people in numerous countries, including Hong Kong [5]. Following concerted efforts by local governments and various community sectors in Hong Kong, the reported number of drug abuse cases fell from 14,241 in 2008 to 4018 in 2018, a decline of 40% [6,7,8]. Despite these seemingly promising figures, hidden drug abuse remains a significant concern in Hong Kong [9]. The most recent figures from the Central Registry of Drug Abuse [10] indicate that the median period spent abusing drugs by newly reported abusers has continued to rise, from 4.6 years in 2017 to 4.7 years in 2018. Multiple factors contribute to hidden drug abuse. First, there is an increasingly stringent legal approach to drug abuse, which can lead to fear of self-disclosure [11]. Second, there has been a shift in drug types, from psychotropic substances such as heroin to so-called less psychotropic substances such as cannabis. Because these less psychotropic substances may result in less readily apparent symptoms of dependence if they are only abused for a very short period of time, the abusers’ motivation to seek help is reduced until prolonged drug abuse results in serious harm to their health or everyday life [11]. Thus, there is an urgent need to develop an appropriate intervention that encourages hidden drug abusers to report their situations and seek professional help.

Developing a community-based network by inviting community members, particularly peers, to refer drug abusers to existing anti-drug services is a common strategy used to identify hidden drug abusers in Western countries [12,13]. There are several reasons for the effectiveness of such networks. First, the rapport that has been established means that hidden drug abusers feel more comfortable disclosing their drug abuse to peers rather than to social workers or healthcare professionals [14]. Second, peers are likely to possess a greater understanding of the psychological and social factors that lead to drug abuse [14]. Although the community-based network has been successful in identifying hidden drug abusers, several difficulties have been encountered during implementation. One of the major difficulties is that the hidden drug abusers are unaware of the importance of ceasing their drug abuse, particularly when they only experience mild dependence symptoms and bodily signs when abusing less psychotropic substances such as cannabis [11]. Another difficulty is that people who are closely connected with hidden drug abusers are usually their friends, and despite their understanding of the importance of ceasing the drug abuse, they find it difficult to refer the abusers to existing anti-drug services. A majority of them do not fully understand the referral processes, are worried about the legal consequences following referral and sometimes even fear that the hidden drug abusers might be forcibly institutionalized to receive mandatory treatment [15]. These misunderstandings often prevent them from encouraging hidden drug abusers to whom they are closely connected to seek professional help [15]. Given these problems, it is of paramount importance to enhance the ability of the community-based network to refer hidden drug abusers to treatment services.

Based on our previous experience involving smoking-cessation trials [16,17], we observed that providing training for peers to serve as anti-drug ambassadors (ADAs) might be a feasible approach to improving the community-based network. Hence, equipping peers with necessary knowledge, as well as changing their attitudes and practice regarding drug abuse, in training is a necessary steps to enable them to be involved in the community-based network as ambassadors. In particular, after training, ADAs can act as a positive role model and disseminate anti-drug knowledge among their peers, especially in relation to the hazardous effects of less psychotropic drugs such as cannabis. In addition, ADAs can better understand the referral process, thereby enhancing their confidence and competence in making referrals when they encounter drug abusers. The Ask, Warn, Advise, Refer and Do-it-again (AWARD) model [18,19,20], which has been widely used in smoking cessation, is also useful for enhancing ADAs’ confidence and competence in relation to making referrals. This model emphasizes the use of a strong warning message to increase people’s awareness of the need to seek help. The advice based on the AWARD model is simple and structured, does not require intensive training and can easily be learned and implemented by laypersons. In addition, the relevant advice can be provided in just a few minutes, which makes it feasible in community settings [20].

To address the existing service gap, we aimed to establish a community-based network to promote anti-drug abuse messaging and identify and refer hidden drug abusers by training peers as ADAs. To evaluate the effectiveness of such network, we examined the change in knowledge, attitudes and practices regarding drug abuse among ADAs before and after the training as the ADAs took the lead to identify and refer hidden drug abusers.

## 2. Materials and Methods

### 2.1. Overview of the Study Design

To develop a community-based network to identify hidden drug abusers by training peers as ADAs, we collaborated with the Community Health Organization for Intervention Care and Empowerment (CHOICE) to conduct a two-phase project in four Hong Kong districts, Kwai Tsing, Northern, Tai Po and Sha Tin. CHOICE is a non-governmental organization that was founded in 2002 with the aim of promoting the prevention of communicable diseases, especially sexually transmitted diseases. Subsequently, CHOICE recognized the need to tackle drug abuse and has provided various counselling services for drug abusers for more than nine years [21]. The main reason for choosing the four abovementioned districts was that they had the highest prevalence of reported drug abuse among those aged under 21 in 2019 [22]. A one-group pre-test and repeated post-test design was used to evaluate the overall effectiveness of the community-based network in promoting anti-drug abuse messaging.

### 2.2. Description of Activities

The study was conducted from November 2020 to April 2022 and was divided into two phases: the establishment of a community-based network by training ADAs and a mass promotional campaign aimed at delivering anti-drug abuse messaging in the targeted districts.

#### 2.2.1. Phase 1: Developing a Community-Based Network by Training ADAs

Phase 1 aimed to develop a community-based network by training ADAs. In this phase, we recruited 150 participants aged 13–18 from secondary schools and community centers in the four targeted districts by sending invitation letters. The content of the training workshop was designed by our expert panel, which consisted of an associate professor, an assistant professor, a research assistant professor, a senior teaching fellow and a senior clinical associate from a university, and the chief executive officer of CHOICE, all of whom had considerable experience in providing counselling services for people with addictive behaviors. The workshop ran for six hours and was delivered by an experienced counsellor trained by our project team. The content of the workshop included (1) the hazardous effects of drug abuse, particularly the abuse of less psychotropic drugs such as cannabis, (2) alternatives to drug abuse, (3) available treatment and rehabilitation services for drug abusers, (4) legal issues related to drug abuse and (5) how to refer drug abusers based on the AWARD model. This model includes five important elements: (1) ask about drug abuse, (2) warn about the hazardous effects of drug abuse, (3) advise the drug abuser to quit immediately (4) refer drug abusers to CHOICE (or another anti-drug organization based on the abuser’s preference) and (5) do-it-again until drug abusers attempt to quit. Those who completed the workshop received a certificate.

#### 2.2.2. Phase 2: A Mass Promotional Campaign Aimed at Delivering Anti-Drug Abuse Messaging in the Community

Phase 2 aimed to encourage the public to join the community-based network through anti-drug activities organized by the ADAs. In addition, we mobilized ADAs to deliver the appropriate advice based on the AWARD model when they encountered drug abusers. Participants who completed the training workshop in Phase 1 were invited to join Phase 2. In Phase 2, the ADAs were encouraged to design and implement their own promotional activities for community members in the targeted districts over a six-month period. The activities could be delivered either by a group (with a maximum of five ADAs per group) or individually. The types of activities included but were not limited to health talks, booths, outreach activities, games, paintings, posters, leaflets and websites. Although the ADAs could design their own promotional activities, they were required to convey at least one of the following messages to community members: (1) the hazardous effects of drug abuse, especially the less psychotropic drugs such as cannabis, (2) encouragement for drug abusers, (3) details of existing anti-drug services and (4) alternatives to drug abuse. Throughout the process, each group of ADAs was required to document their aims, types of activities undertaken, venues and numbers of people reached. They were also invited to reflect on their experiences while undertaking these activities in a logbook they were given.

If the ADAs encountered drug abusers in their social circles, they were asked to provide advice and make a referral based on the AWARD model. Specifically, they were instructed to ask the drug abusers about their drug-taking history such as the types and daily consumption levels of drugs and the number of attempts they had made to quit. They would then deliver the following warning message: “According to the Department of Health, abusing drugs lead to irreversible damage to your brain, liver, lungs, kidneys, and heart. It also destroys your future. Recent medical evidence has shown that the risk of death of drug abusers is 11 times higher than that of the general population. This is very high, and dangerous. You have to stop abusing drugs immediately because doing so is good for you”. If the abuser refused to stop immediately, the ADA would advise them to reduce their daily drug consumption. The ADA would then introduce the services provided by CHOICE and other anti-drug organizations and refer them to either CHOICE or another anti-drug organization based on the abuser’s preference after obtaining their permission. If they encountered the same drug abuser again, the ADAs would repeat the above actions until the abuser ceased their drug abuse.

### 2.3. Participants

The most recent figures published by the Central Registry of Drug Abuse indicate that 41.1% of newly reported abusers first took drugs at the age of 16–20, while 37.4% first took drugs before the age of 16 [23]. It is anticipated that these drug abusers started their drug abuse behaviors in adolescence. Thus, it is crucial for healthcare professionals to implement preventive measures targeting this age group. Therefore, in this study, we recruited participants aged 13–18.

Since this was a health promotion project, the number of ADAs to be trained was calculated based on the amount of funding as well as the funding period. However, this sample size was comparable with those in other projects that assess the change in knowledge, attitudes and practices before and after training among different populations [16,17].

### 2.4. Outcomes

To examine the overall effectiveness of the community-based network, we evaluated the outcomes of the participants, all of whose parents had signed consent forms. The outcome measures were (1) knowledge about the hazardous effects of drug abuse, (2) attitudes toward drug abuse and (3) anti-drug practices.

### 2.5. Data Collection

A structured questionnaire designed by the Narcotics Division of the Security Bureau, Hong Kong Special Administrative Region [24], and modified by our project team was administered to the participants in Phase 1 at baseline, immediately after the training workshop, and three and six months later to assess the changes in their knowledge of and attitudes toward drug abuse. In addition, the participants were asked to report on their anti-drug practices using the questionnaires at baseline and three and six months after the training workshop.

For Phase 2, we recorded the numbers and types of activities organized by the ADAs, the numbers of people who were reached by the activities and the numbers of drug abusers being referred to CHOICE and other anti-drug organizations during the six-month period following the training.

### 2.6. Data Analysis

The Statistical Package for the Social Sciences (SPSS: Version 26; SPSS Inc., Chicago, IL, USA) for Windows was used to analyze the data. Descriptive statistics were used to summarize the participants’ demographic characteristics. One-way repeated-measures ANOVA was used to assess the changes in the participants’ knowledge of and attitudes toward drug abuse, as well as their anti-drug practices before and after the training workshop. Bonferroni correction was used as the post hoc test, with the alpha level (α = 0.05) being adjusted by dividing the number of tests running. The normality of continuous data was confirmed by Q-Q plots visually and Kolmogorov–Smirnov test statically. All the data were shown to be normally distributed and fulfilled the statistical assumption of ANOVA. Descriptive statistics were also used to describe the activities organized by the ADAs.

### 2.7. Ethical Approval and Consent

Prior to the commencement of this study, ethical approval was obtained from the Human Subjects Ethics Sub-committee of The Hong Kong Polytechnic University (#HSEARS20200304002). The study was also registered at clinicaltrial.gov (#NCT04400526). Because the participants were no older than 18, written consent was obtained from their parents after they were fully informed about the study’s purpose and details.

## 3. Results

Between November 2020 and April 2022, we recruited a total of 256 secondary school students to receive the training and serve as ADAs. However, 8.9% (*n* = 23) and 13.7% (*n* = 35) were lost to follow-up at three and six months, respectively. Hence, only 198 ADAs were included in our analysis.

The baseline characteristics of the participants are presented in Table 1. Of the ADAs, whose mean age was 14.7 years (SD = 1.37), 55.6% (*n* = 110) were female, none were drug abusers and 1.6% (*n* = 3) reported that they had a drug abuser in their social circle, one of whom was a family member, while the other two who were friends.

Table 2 shows the changes in the ADAs’ knowledge regarding drug abuse during the evaluation period. It can be seen that most ADAs showed significant improvement in terms of their knowledge regarding drug abuse, with the *p*-values adjusted to <0.001 to achieve statistically significant according to Bonferroni correction. Specifically, more ADAs agreed with the following statements: “Taking drugs can make you look ugly” (*p* < 0.001); “Drug abuse impairs judgment” (*p* < 0.001); “Drug abuse can lead to a decline in thinking ability and memory” (*p* < 0.001); “Cannabis is illegal in Hong Kong” (*p* < 0.001); “Cannabis use can lead to dependence” (*p* < 0.001); “Cannabis use can expose people to increased health risks related to their brain, heart and lungs” (*p* < 0.001); “Long-term use of cannabis or over-consumption of cough medicine may lead to auditory hallucinations and delusions” (*p* < 0.001); “Taking ketamine-based drugs can damage the kidneys and bladder” (*p* < 0.001); “Taking certain drugs can lead to insanity and even psychosis” (*p* < 0.001); “Ketamine-based drugs are often mixed with other harmful substances, causing more serious damage” (*p* < 0.001); “Taking ice-based drugs can cause skin rashes, leading to ice sores” (*p* < 0.001); and “Taking heavy doses of ice can lead to psychosis, convulsions, coma, cerebral hemorrhage and even death” (*p* < 0.001). However, no significant improvement was found in relation to the statement “Certain drugs can help you lose weight” (*p* = 0.447).

Table 3 shows the changes in the ADAs’ attitudes toward drug abuse during the evaluation period, and the *p*-values were adjusted to be <0.001 to attain statistical significance based on Bonferroni correction. It can be seen that there were significant improvements in terms of attitudes toward drug abuse following the training. In particular, more ADAs agreed with the following statements: “Drug abuse can impair memory and judgment” (*p* < 0.001); “Even if you have only taken drugs once, you can still get addicted” (*p* < 0.001); “Many drugs cause irreparable damage to the body” (*p* < 0.001); “If I take drugs, my daily life and my studies will be affected” (*p* < 0.001); “Under the influence of drugs, the body is easily abused or damaged” (*p* < 0.001); “Under the influence of drugs, psychotic symptoms such as auditory hallucinations or delusions can occur” (*p* < 0.001); “Under the influence of drugs, it is easy to engage in risky activities” (*p* < 0.001); “Under the influence of drugs, it is easy to say hurtful words to loved ones” (*p* < 0.001); “Under the influence of drugs, it is easy to do stupid things impulsively that you will regret later” (*p* < 0.001); “Under the influence of drugs, it is easy to engage in life-threatening behaviors (*p* < 0.001); “Everyone who has ever taken drugs eventually regrets it” (*p* < 0.001); and “Nothing is more dangerous than taking drugs” (*p* < 0.001).

Table 4 shows the changes in the ADAs’ anti-drug-abuse practices during the evaluation period. It can be seen that there was a significant increase in the mean self-efficacy score in terms of referring drug abusers six months after the training compared with the baseline (*p* < 0.001), with the *p*-value adjusted to be <0.001 to be statistically significant according to Bonferroni correction. All three ADAs who had drug abusers in their social circle reported delivering brief advice and making a referral based on the AWARD model at the three- and six-month follow-ups. Of the other ADAs who did not report making any referrals at the six-month follow-up, most (*n* = 182/195, 93.3%) reported that they did not know any drug abusers. Other reasons given were that they did not think it was their responsibility (*n* = 50/195, 25.6%) and that they perceived that referral was not effective (*n* = 45/195, 23.1%).

Table 5 shows the anti-drug abuse promotional activities organized by the ADAs during the evaluation period. A total of 154 anti-drug promotional activities were organized by the ADAs. The activities were diverse in nature and included videos (*n* = 68), posters (*n* = 42), promotional booths (*n* = 8), WhatsApp recordings (*n* = 30) and other activities (*n* = 6). These activities enabled the ADAs to disseminate the anti-drug message to 4561 people in secondary schools and the community.

## 4. Discussion

Hidden drug abuse remains a significant concern for public health. This study aimed to enhance the community’s capacity to identify and refer hidden drug abusers by training peers as ADAs. In addition, using the promotional activities developed by the ADAs, we aimed to widely disseminate the anti-drug message in schools and the community.

Our results based on repeated-measures ANOVA showed that the training program was effective in enhancing the ADAs’ knowledge regarding the negative health effects of drug abuse after the *p*-values were adjusted by Bonferroni correction. Specifically, there was a significant improvement in the ADAs’ knowledge regarding the damaging effects of various drugs such as cannabis, ketamine, cough medicine and ice on cognitive functions (*p* < 0.001), mental health (*p* < 0.001) and outward appearance (*p* < 0.001), as well as the kidneys and bladder (*p* < 0.001) and skin (*p* < 0.001). However, there was no significant change in the ADAs’ understanding of whether drug abuse can help with weight reduction (*p* = 0.477). One possible explanation was that at baseline, a majority of the ADAs already knew that drug abuse was not an appropriate method of losing weight, and thus we were unable to detect any significant improvement in their knowledge in this regard. The results also showed that the training program was effective in changing the attitudes of the ADAs toward drug abuse, in particular enhancing their perceptions regarding the negative effects of drug abuse on personal health and daily life, as well as regret about taking drugs. Since 2018, there has been an upsurge in drug abuse among people aged 20 or below in Hong Kong [25], and in 2021, the mean age at which the drug abuse commenced was 16 for males and 15 for females [26]. These figures concur with the results of a nationwide survey in the United States, which showed that the median age of initial substance use was approximately 15, depending on ethnicity and substance type [27]. There is considerable evidence indicating that improved knowledge and attitudes regarding drug abuse increase adolescents’ self-efficacy in relation to abstaining from such behavior [28,29]. Therefore, it is expected that our ADAs will not engage in drug-taking behavior following their training and will continue to portray a positive image among their peers.

There was also a significant improvement in the ADAs’ self-efficacy in terms of referring drug abusers to anti-drug services (*p* < 0.001) after the *p*-values were adjusted by Bonferroni correction. In addition, all of the ADAs with drug abusers in their social circle indicated that they had followed the AWARD model in referring the abusers to professional help. However, the data indicated that only three ADAs used the AWARD model during the evaluation period, with only one drug abuser being successfully referred at the time of the six-month follow-up. Thus, caution must be exercised when interpreting these figures because they were highly confounded by the number of drug abusers encountered by the ADAs at baseline and throughout the evaluation period. Because the study was conducted during the COVID-19 pandemic, all secondary schools in Hong Kong were closed, significantly reducing the level of social contact among students and the possibility of the ADAs identifying hidden drug abusers in their social circles. Furthermore, the number of drug abusers remains a very small proportion of Hong Kong’s total population. Hence, our ADAs might not have had any friends who were hidden drug abusers and could be referred. This is supported by the fact that only three of the total of 198 ADAs reported knowing drug abusers at baseline. Thus, to evaluate the overall effectiveness of the community-based network in identifying and referring hidden drug abusers, it is recommended that our training program be conducted on a larger scale with more students being trained as ADAs.

Between December 2021 and June 2022, our trained ADAs organized a total of 154 activities that reached 4561 people in secondary schools and the community. This was a remarkable achievement given the impact of the COVID-19 pandemic, with approximately 30 people on average being reached by each activity. The activities undertaken by the ADAs varied in nature and included some that were temporary such as promotional booths and some that were more permanent such as YouTube videos. Although the study has ended, it is expected that these activities will have a snowballing effect and serve as a practical and effective way to continuously disseminate anti-drug messages to a large number of people. The cost of launching these activities was low compared with healthcare expenditure on mortality and morbidity associated with drug abuse. Thus, the activities undertaken by our ADAs could have a substantial impact in terms of saving lives and reducing healthcare expenditure if they could be extended to all other districts in Hong Kong.

During our interactions with the secondary school students and their teachers in the training program, quite a number of them said that they had heard about “cannabis restaurants” and “cannabis drinks and foods” via friends and social media platforms, including Facebook and Instagram. Perhaps this could be attributed to an exponential increase in recent years in the use of cannabidiol (CBD) products for health and wellness in Western countries [30] and Hong Kong [31]. Differing from the psychoactive compound tetrahydrocannabinol (THC), CBD is another key ingredient of cannabis that in principle is not psychoactive and thus does not have the potential for abuse [32]. As a marketing strategy, businesses have added CBD to various types of foods and drinks and advertised these products as “cannabis-based” or “cannabis-related” [33,34], which appeals to young people who like to experiment with products whose legal status is unclear [35]. These CBD products are also advertised as providing various health benefits such as alleviating anxiety and chronic pain [36], thereby attracting people who are inclined to pursue non-pharmacological treatments for their conditions. Although CBD is claimed to have some therapeutic effects for various medical conditions and appears to be a lot safer than THC, public perceptions of the worth of CBD exceed its clinical effectiveness for many of the conditions for which it is currently used. The US Food and Drug Administration states that CBD products are generally marketed using unproven medical claims and are of unknown quality [37]. For example, prolonged use of CBD can lead to changes in alertness, by promoting drowsiness or sleepiness, and fluctuations in moods such as increased irritability and agitation [37]. CBD can also interact with other drugs, resulting in severe side effects [37]. CBD can be extracted from cannabis, and thus CBD products can contain very low levels of THC, which remains psychoactive [38]. A survey of 15,042 people in Canada and 30,288 people in the US aged 16–65 revealed that approximately 16% and 26% of respondents in Canada and the US, respectively, had used CBD products during the previous year [39]. One alarming finding from this survey is that CBD product use was shown to be more prevalent among those who reported more frequent cannabis use [39]. This suggested that CBD use might promote the use of cannabis. To combat these upcoming challenges, health education should focus on clarifying misconceptions among secondary school students regarding the therapeutic effects of CBD. In addition, policy-makers should intervene to at least control or even ban the use of CBD products among young people who are unable to differentiate between CBD and THC.

### Limitations

Firstly, this study was conducted during the COVID-19 pandemic, when all schools in Hong Kong were closed. Although some secondary schools acknowledged the importance of our training program, they were reluctant to participate because of the uncertainty surrounding the pandemic. Hence, only a few secondary schools agreed to participate in the study, and it is unclear whether the reluctance of other schools to participate was because of concerns about the study or the impact of the COVID-19 pandemic. Secondly, the evaluation period only extended for six months after the training workshop, and thus the long-term impact of our training program on the ADAs, in particular their drug abuse behavior, is unknown. Thirdly, the sample size was only calculated based on the available funding as well as the funding period, but not statistical methods, thus resulting in a type II error. It is recommended that future studies use statistical methods to calculate the required sample size.

## 5. Conclusions

The community-based network was found to be effective in disseminating anti-drug-use messaging among secondary school students and the community. The results of our study showed that the involvement of our ADAs in community-based networks led to significant improvements in their knowledge of and attitudes toward drug abuse, as well as their self-efficacy in terms of referring drug abusers to anti-drug services. On the basis of these promising results, we believe that this community-based network should be implemented on a larger scale, thereby increasing our ability to identify hidden drug abusers.

## Figures and Tables

**Table 1:** Baseline characteristics of anti-drug ambassadors (*n* = 198).

Sex, *n* (%)	
Male	88 (44.4)
Female	110 (55.6)
Age, mean (SD)	14.7 (1.37)
Drug abuse status, *n* (%)	
Non-drug abuser	198 (100.0)
Current drug abuser	0 (0)
Ex-drug abuser	0 (0)
Having drug abusers in the social circle, *n* (%)	
No	195 (98.4)
Yes	3 (1.6)
Relationship with the drug abusers, *n* (%)	
Family member	1/3 (33.3)
Friend	2/3 (66.7)

Note: SD = Standard deviation; *n* = number of participants in the denominator.

**Table 2:** Change in knowledge towards drug abuse among anti-drug ambassadors by repeated-measures ANOVA (*n* = 198).

	T1	T2	T3	T4	*F*-Value	*p*-Value
1. Taking drugs can make you look ugly, mean (SD)	4.17 (0.84)	4.64 (0.59)	4.59 (0.62)	4.59 (0.66)	76.28	<0.001 *
2. Drug use impairs judgment, mean (SD)	4.39 (0.80)	4.66 (0.54)	4.62 (0.62)	4.58 (0.67)	26.85	<0.001 *
3. Drug use can lead to a decline in thinking ability and memory, mean (SD)	4.39 (0.80)	4.67 (0.55)	4.60 (0.67)	4.56 (0.72)	24.02	<0.001 *
4. Cannabis in Hong Kong is illegal, mean (SD)	4.00 (0.94)	4.65 (0.57)	4.55 (0.63)	4.45 (0.63)	66.76	<0.001 *
5. Cannabis use can lead to dependence, mean (SD)	3.75 (1.06)	4.71 (0.51)	4.62 (0.56)	4.54 (0.66)	102.37	<0.001 *
6. Cannabis use can expose people to increased health risks for the brain, heart and lungs, mean (SD)	3.90 (1.05)	4.52 (0.66)	4.44 (0.71)	4.38 (0.77)	37.60	<0.001 *
7. Long-term use of cannabis or over-consumption of cough medicine may lead to auditory hallucinations and delusions, mean (SD)	4.34 (0.83)	4.68 (0.54)	4.59 (0.68)	4.55 (0.68)	31.25	<0.001 *
8. Taking ketamine-based drugs can damage the kidneys and bladder, mean (SD)	4.28 (0.86)	4.67 (0.52)	4.58 (0.67)	4.55 (0.67)	36.33	<0.001 *
9. Certain drugs can help you lose weight, mean (SD)	2.59 (1.17)	2.45 (1.38)	2.47 (1.42)	2.51 (1.44)	0.51	0.477
10. Taking certain drugs can lead to insanity and even psychosis, mean (SD)	4.25 (0.82)	4.63 (0.55)	4.57 (0.66)	4.53 (0.70)	39.18	<0.001 *
11. Ketamine-based drugs are often mixed with other harmful substances, causing more serious damage, mean (SD)	4.20 (0.88)	4.65 (0.56)	4.59 (0.67)	4.57 (0.69)	54.52	<0.001 *
12. Taking “Ice”-based drugs can cause skin rashes, leading to ice sores, mean (SD)	4.04 (0.91)	4.66 (0.58)	4.59 (0.68)	4.56 (0.72)	79.04	<0.001 *
13. Taking heavy doses of “Ice” can lead to psychosis, convulsions, coma, cerebral hemorrhage and even death, mean (SD)	4.20 (0.86)	4.64 (0.56)	4.59 (0.65)	4.57 (0.67)	53.47	<0.001 *

Note: Range of scores = 1 (strongly disagree) to 5 (strongly agree); SD = Standard deviation; T1 = before training, T2 = immediately after training; T3 = 3 months after the training; T4 = 6 months after the training; the Bonferroni correction was used to adjust *p*-value; * = adjusted *p*-value < 0.01.

**Table 3:** Change in attitudes towards drug abuse among anti-drug ambassadors by repeated-measures ANOVA (*n* = 198).

	T1	T2	T3	T4	*F*-Value	*p*-Value
1. Drug use can impair memory and judgment, mean (SD)	4.36 (0.78)	4.67 (0.53)	4.63 (0.58)	4.60 (0.59)	37.84	<0.001 *
2. Even if you have only taken drugs once, you can still get addicted, mean (SD)	4.19 (0.91)	4.58 (0.61)	4.51 (0.70)	4.48 (0.70)	36.70	<0.001 *
3. Many drugs cause irreparable damage to the body, mean (SD)	4.28 (0.84)	4.64 (0.54)	4.58 (0.64)	4.54 (0.65)	37.27	<0.001 *
4. If I take drugs, my daily life and my studies will be affected, mean (SD)	4.41 (0.80)	4.68 (0.50)	4.64 (0.59)	4.59 (0.64)	23.47	<0.001 *
5. Under the influence of drugs, the body is easily abused or damaged, mean (SD)	4.20 (0.93)	4.63 (0.57)	4.57 (0.60)	4.47 (0.71)	37.86	<0.001 *
6. Under the influence of drugs, psychotic symptoms such as auditory hallucinations or hallucinations may occur, mean (SD)	4.39 (0.77)	4.66 (0.52)	4.61 (0.59)	4.58 (0.59)	25.54	<0.001 *
7. Under the influence of drugs, it is easy to engage in risky activities, mean (SD)	4.28 (0.87)	4.63 (0.56)	4.55 (0.67)	4.50 (0.67)	28.34	<0.001 *
8. Under the influence of drugs, it is easy to say hurtful words to loved ones, mean (SD)	4.16 (0.94)	4.56 (0.71)	4.49 (0.79)	4.43 (0.79)	37.90	<0.001 *
9. Under the influence of drugs, it is easy to do stupid things impulsively that you will regret later, mean (SD)	4.38 (0.80)	4.67 (0.53)	4.60 (0.64)	4.54 (0.67)	24.28	<0.001 *
10. Under the influence of drugs, it is easy to engage in life-threatening behaviors, mean (SD)	4.33 (0.84)	4.68 (0.51)	4.60 (0.65)	4.56 (0.69)	30.02	<0.001 *
11. Everyone who has ever taken drugs eventually regrets, mean (SD)	3.71 (0.97)	4.41 (0.77)	4.36 (0.78)	4.32 (0.82)	105.46	<0.001 *
12. Nothing is more dangerous than taking drugs, mean (SD)	3.67 (1.03)	4.31 (0.93)	4.24 (0.98)	4.18 (1.02)	32.68	<0.001 *

Note: Range of scores = 1 (strongly disagree) to 5 (strongly agree); SD = Standard deviation; T1 = before training, T2 = immediately after training; T3 = 3 months after the training; T4 = 6 months after the training; the Bonferroni correction was used to adjust *p*-value; * = adjusted *p*-value < 0.01.

**Table 4:** Changes in anti-drug practices among anti-drug ambassadors (*n* = 198).

	T1	T2	T3	T4	*F*-Value	*p*-Value
Number of anti-drug ambassadors who know drug abusers in their social circle, *n* (%)	3 (1.52)	-	3 (1.52)	3 (1.52)	-	-
Of anti-drug ambassadors who know drug abusers (*n* = 3)						
Referring drug abusers to treatment and rehabilitation service in the past 3 months, *n* (%)	0 (0)	-	3 (100)	3 (100)	-	-
Number of drug abusers being successfully referred, *n* (%)	0 (0)	-	0 (0)	1 (33.3)	-	-
Reasons for anti-drug ambassadors who do not make referral (*n* = 195) ^a^						
Don’t know any drug abusers, *n* (%)	171 (87.7)	-	178 (91.3)	182 (93.3)	-	-
I don’t know any available treatment and rehabilitation service, *n* (%)	145 (74.4)	-	32 (16.4)	35 (17.9)	-	-
I don’t have the skills to make referral, *n* (%)	156 (80.0)	-	40 (20.5)	38 (19.5)	-	-
I don’t want to harm the relationship, *n* (%)	98 (50.2)	-	45 (23.1)	32 (16.4)	-	-
I worry that the drug abusers may have legal consequences after being referred to treatment and rehabilitation service, *n* (%)	72 (36.9)	-	36 (18.5)	43 (22.1)	-	-
I don’t think the referral effective, *n* (%)	112 (57.4)	-	49 (25.1)	45 (23.1)	-	-
I don’t have the responsibility, *n* (%)	90 (46.1)	-	43 (22.1)	50 (25.6)	-	-
Perceived self-efficacy in referring drug abusers ^b^ (1 = “completely not” to 4 = “absolutely true”) (*n* = 198)						
I am confident in referring drug abusers to treatment and rehabilitation service, mean (SD)	2.56 (1.12)	3.63 (0.62)	3.48 (0.66)	3.43 (0.65)	135.21	<0.001 *
I don’t have any difficulty in referring drug abusers to treatment and rehabilitation service, mean (SD)	3.42 (0.88)	2.56 (1.09)	2.63 (1.05)	2.70 (1.03)	125.05	<0.001 *
This is important to refer drug abusers to treatment and rehabilitation service, mean (SD)	2.40 (0.83)	3.40 (0.92)	3.23 (0.88)	3.17 (0.86)	169.35	<0.001 *
Overall mean score	2.18 (0.49)	3.16 (0.48)	3.03 (0.47)	2.97 (0.47)	394.24	<0.001 *

Note: T1 = before training, T2 = immediately after training; T3 = 3 months after the training; T4 = 6 months after the training; ^a^ = can indicate more than one answer; ^b^ = repeated-measures ANOVA was used; the Bonferroni correction was used to adjust *p*-value; * = adjusted *p*-value <0.01.

**Table 5:** Promotional activities organized by anti-drug ambassadors.

Number of promotional activities organized	154
Types of promotional activities, *n* (%)	
Video production	68/154 (44.2)
Poster design	42/154 (27.3)
Booth promotion	8/154 (5.2)
WhatsApp recording	30/154 (19.5)
Others	6/154 (3.9)
Number of people reached by the activities	4561

## Data Availability

Data will be available upon reasonable request.
